# Physicians’ perspective on shared decision-making in Dubai: a cross-sectional study

**DOI:** 10.1186/s12960-020-00475-x

**Published:** 2020-05-07

**Authors:** Mohamad Alameddine, Reem AlGurg, Farah Otaki, Alawi A. Alsheikh-Ali

**Affiliations:** 1 Department of Health Management and Policy, Faculty of Health Sciences, American University of Beirut, Beirut, Lebanon; 2 College of Medicine, Mohammed Bin Rashid University of Medicine and Health Sciences, P.O. Box 505055, Dubai, United Arab Emirates; 3 Strategy and Institutional Excellence, Mohammed Bin Rashid University of Medicine and Health Sciences, Dubai, United Arab Emirates

**Keywords:** Shared decision-making, Outpatient clinics, Physicians, Dubai

## Abstract

**Background:**

Shared decision-making (SDM) is an integral part of patient-centered delivery of care. Maximizing the opportunity of patients to participate in decisions related to their health is an expectation in care delivery nowadays. The purpose of this study is to explore the perceptions of physicians in regard to SDM in a large private hospital network in Dubai, United Arab Emirates.

**Methods:**

This study utilized a cross-sectional design, where a survey questionnaire was assembled to capture quantitative and qualitative data on the perception of physicians in relation to SDM. The survey instrument included three sections: the first solicited physicians’ personal and professional information, the second entailed a 9-item SDM Questionnaire (SDM-Q-9), and the third included an open-ended section. Statistical analysis assessed whether the average SDM-Q-9 score differed significantly by gender, age, years of experience, professional status—generalist versus specialist, and work location—hospitals versus polyclinics. Non-parametric analysis (two independent variables) with the Mann-Whitney test was utilized. The qualitative data was thematically analyzed.

**Results:**

Fifty physicians from various specialties participated in this study (25 of each gender—85% response rate). Although the quantitative data analysis revealed that most physicians (80%) rated themselves quite highly when it comes to SDM, qualitative analysis underscored a number of barriers that limited the opportunity for SDM. Analysis identified four themes that influence the acceptability of SDM, namely physician-specific (where the physicians’ extent of adopting SDM is related to their own belief system and their perception that the presence of evidence negates the need for SDM), patient-related (e.g., patients’ unwillingness to be involved in decisions concerning their health), contextual/environmental (e.g., sociocultural impediments), and relational (the information asymmetry and the power gradient that influence how the physician and patient relate to one another).

**Conclusions:**

SDM and evidence-based management (EBM) are not mutually exclusive. Professional learning and development programs targeting caregivers should focus on the consolidation of the two perspectives. We encourage healthcare managers and leaders to translate declared policies into actionable initiatives supporting patient-centered care. This could be achieved through the dedication of the necessary resources that would enable SDM, and the development of interventions that are designed both to improve health literacy and to educate patients on their rights.

## Background

Shared decision-making (SDM) is a cornerstone of patient-centered delivery of care. It involves a set of interactions between the patient and the healthcare provider to ensure high-quality decisions in managing patient care [1]. SDM involves discussing the health problem with the patient, while presenting medically appropriate treatment options, and in turn, selecting the best evidence-driven choice, while considering the goals, preferences, and concerns of the respective patient [1]. Accordingly, patients engaged in SDM are fully informed throughout the decision-making process and actively participate in selecting the best possible treatment option [1, 2].

Studies have shown that SDM improves health outcomes [3–5], patients’ adherence to treatment [3], and patient satisfaction with decisions [6] and with the overall quality of care [7], while reducing healthcare costs [3]. SDM can also allow for the adoption of treatments that better suit the lifestyle and personal preferences of the patient [8]. Since decision-making in healthcare is becoming more complex due to the availability of different treatment options with possibly varied health outcomes [9], the adoption of SDM has not only been encouraged but is also becoming an ethical mandate of the medical profession [10]. Therefore, enhancing patient-centered care through the promotion of SDM in clinical settings is now considered among the top priorities for health managers and policymakers in healthcare systems around the globe.

Despite the aforementioned advantages of SDM, many healthcare providers are still minimally engaging patients in decisions related to their health [2]. This is partly because it takes time to enable effective negotiation and communication between the healthcare provider and the patient [8]. In fact, for some providers, SDM is hard to do and is often not taught during medical training [11]. Reported barriers to SDM implementation include high workload, patients’ characteristics, and the clinical situation [11]. Furthermore, physicians may not support SDM in situations where less treatment options were available; guidelines stipulated only one treatment option, in emergency cases; and/or when the patient was unable or unwilling to participate in decision-making [2].

Favorable attitudes and behaviors toward SDM among healthcare providers are pivotal for the delivery of proper and equitable patient-centered care [2]. Reported facilitators include the healthcare provider’s motivation and positive effects on the clinical process and the health outcomes [12].

According to Kon [13], the extent of patient engagement in decision-making can be anywhere on a continuum that ranges from an evidence-based decision made by the physician, on his/her own, all the way to a patient-driven interaction. Sociocultural factors (e.g., value system, attitude, norms, age, educational background, and preferred language) may either facilitate or inhibit the entailed information exchange [14]. The importance of addressing those sociocultural variables in order to raise the extent of patient engagement in decision-making is established in the literature [15, 16]. Although there are several studies that assess the positioning of clinical encounters on the SDM continuum, very few capture the physicians’ perspective and most of them were conducted in the Western world. In order to increase the adoption of SDM in the Middle East region, there needs to be a more context-specific understanding of physicians’ perspective. Accordingly, the purpose of this study is to explore the perception in relation to SDM of physicians working in a large private healthcare delivery network in Dubai, United Arab Emirates (UAE).

## Methods

### Research design

This study utilized a cross-sectional design, where a survey questionnaire was assembled to capture quantitative and qualitative data on the perception of physicians in relation to SDM. It is worth noting that this study reports on the results of Stage One of a larger research work examining whether the concordance versus discordance of gender of the physician and patient affects the extent of SDM during outpatient clinic consultation.

### Context of the study

The study was carried out in Dubai, the largest and most populous of the seven Emirates of the UAE. Dubai is a major tourist destination and global hub for trade, industry, and economy. The population of the city is characteristically diverse, with Dubai hosting more than 200 nationalities from all around the globe [17].

The delivery of healthcare in Dubai is a shared public and private responsibility. It is estimated that around 70% of the healthcare facilities in Dubai are private and the remaining 30% are public [18].

This study involved physicians practicing in a large private healthcare delivery network (two hospitals, one specialized polyclinic, and four multi-specialization polyclinics) in Dubai. The network, considered among the largest in the UAE, has a capacity of approximately 1000 inpatient beds and around 6000 employees. The network offers a full range of preventive and curative services including primary, secondary, tertiary, and quaternary [19]. The network’s providers and patients are as diverse as the city, coming from various countries and care systems [19].

### Ethical and administrative approvals

The study protocol was cleared from four different IRB boards (details under the “Declarations” section). In addition, administrative approval for data collection was secured from the healthcare provider network under investigation, and a written informed consent was obtained from each of the participating physicians. All approvals were secured prior to the commencement of data collection.

### Survey

The survey questionnaire is composed of three sections. The first section solicits personal and professional data about the physician (gender, age, nationality, year of experience since attainment of highest educational degree, and specialty degree country), as well as information about the clinic in which the respective physician practices/operates (type of clinic: hospital-based, polyclinic, primary healthcare center, and private practice, and specialty). The second section is based on the validated 9-item Shared Decision-Making Questionnaire (SDM-Q-9) [20]. The SDM-Q-9 yields an average score ranging from 1 (indicative of absence of perceived shared decision) to 5 (indicative complete perceived shared decision). The third section of the questionnaire is composed of an open-ended question to solicit qualitative data, where the participant is given unlimited space to provide any relevant additional comment(s) that comes to their mind.

### Pilot study

The survey was piloted on four, randomly selected physicians (two female and two male). Prior to the pilot, the study was meant to be purely deductive, where the physicians’ perception of the SDM was going to be assessed in relation to the differing characteristics of the physicians and the clinic in which they operate as the independent variables. No changes were made to the quantitative part of the questionnaire as a result of the pilot. The open-ended section of the questionnaire, however, was added since the physicians, who participated in the pilot, seemed eager to voice their opinion. The researchers agreed that adding a qualitative component will generate valuable content that is worth inductively analyzing to develop a better understanding of the relevant aspects of patient experience related to SDM.

### Sampling technique

A complete listing of all physicians working in the healthcare delivery network was acquired. Then, a list of all physicians meeting the inclusion criteria was generated. To meet the inclusion criteria, study participants needed to be licensed to practice in Dubai, providing care in specialties that serve both genders (obstetrician-gynecologist, for example, were excluded since they only serve female patients). The inclusion of physicians who only serve both genders is by virtue of the design of the mother study which was mentioned under the “Research design” section. Pediatricians were also excluded (since the study is examining SDM in patients and not in legal guardians). Physicians meeting inclusion criteria were stratified by gender and specialty.

A total of 25 female and 25 male physicians, who meet the inclusion criteria, were randomly selected and invited to participate in the study. If physicians were not available (e.g., were on a vacation) or if they did not agree to participate, they were replaced by another randomly selected physician of the same gender and specialty from the list of the remaining physicians, who meet the inclusion criteria. This process of recruitment continued until the target sample size of 50 physicians (25 female and 25 male) was attained.

A total of 59 physicians (31 female and 28 male) were invited to participate, and 50 responded (85% response rate). Non-participating physicians were either busy (schedule, time constraints, and competing priorities), not interested in research, or not sure the study was anonymous (despite reviewing the consent form and getting assurances of the protection of their privacy and confidentiality).

### Data collection and coding

Data collection took place from July 2018 to October 2018. The physicians were interviewed in their respective clinics by the Field Surveyor, at an agreed upon timing. Each physician agreeing to participate was requested to sign a consent form prior to the initiation of data collection. Questionnaires took an average of around 15 min to complete.

Each participant was referred to with a code that constitutes a numerical value sandwiched in between two letters. The first letter is “P”—an abbreviation of the word “Participant.” The numerical value is unique to each participant (i.e., 1 to 50), and the last letter is either “F” or “M” referring to the gender of the participant (female or male, respectively).

### Quantitative data analysis

The quantitative data was analyzed by three researchers with experience in undertaking quantitative and qualitative research studies. The quantitative analysis was done using SPSS for Windows version 23.0. Analysis utilized a non-parametric (two independent variables) Mann-Whitney test. Significance was set at *P* value less than 0.05. Analysis assessed whether the average SDM-Q-9 score differed significantly by gender, age (< 50 versus ≥ 50), years of experience (< 20 versus ≥ 20), generalist versus specialist, and hospital versus polyclinic.

### Qualitative data analysis

The qualitative data, retrieved from the open-ended section of the questionnaire, was inductively analyzed using thematic analysis by two researchers. This type of qualitative analysis constitutes a systematic way of identifying, organizing, and offering insights into patterns of meaning (themes) across a dataset [21]. Accordingly, this method is a way of identifying what is common to the means by which a topic is referred to, and (more importantly) to shed light on the patterns that are relevant to answering the research question. The reliance on such data analyses techniques is of added value to the theory and practice of the subject matter, offering the discipline a lot of worthwhile insights [22, 23].

The qualitative analysis was initiated by reading the transcripts for all interviews which led to the emergence of several themes. The encapsulated data segments within each of the themes were further investigated to identify a way to identify sub-themes. Finally, the themes were thoroughly reflected upon, and different ways by which these themes relate to one another were identified, based on our understanding of the context and of the dynamics in the local healthcare ecosystem. The researchers also assessed the concordance (vs. discordance) of the quantitative and qualitative findings.

## Results

### Study participants

As Table 1 reveals, out of 50 participants, half were females and the other half were males (as per the research design); 28 are less than 50 years old. In terms of years of experience after attainment of last degree, most physicians have a career tenure of less than 20 years (58%). The majority of the surveyed physicians are specialists (82%). Most of the respondents were hospital-based (41 physicians—82%); the remaining practice in/operate out of polyclinics.

**Table 1:** Description of the study sample

**Characteristic**	**Level**	**Number (%)**
Gender	Female	25 (50.0)
	Male	25 (50.0)
Age (years)	< 50	28 (56.0)
	> 50	22 (44.0)
Years of experience (after attainment of last degree)	< 20	29 (58.0)
	> 20	21 (42.0)
Specialty	Specialist	41 (82.0)
	• Dermatology	1 (2.0)
	• Neurosurgery	1 (2.0)
	• Plastic surgery	1 (2.0)
	• Vascular surgery	1 (2.0)
	• Endocrinology	2 (4.0)
	• Internal medicine	2 (4.0)
	• Nephrology	2 (4.0)
	• Neurology	2 (4.0)
	• General surgery	3 (6.0)
	• Orthopedics	3 (6.0)
	• Rheumatology	3 (6.0)
	• Ear, nose, and throat	4 (8.0)
	• Gastroenterology	5 (10.0)
	• Oncology	5 (10.0)
	• Ophthalmology	6 (12.0)
	Generalist	9 (18.0)
Type of healthcare delivery system	Polyclinic	9 (18.0)
	Hospital	41 (82.0)

### Quantitative analysis output

As revealed in Table 2, the study revealed no significant difference across all independent variables. Most of the physicians (80%) rated themselves quite highly when it comes to SDM.

**Table 2:** Output of quantitative analysis

Independent variables		
**Gender**	**Male**	**Female**
***N***	25	25
**Median score (IQR)**	4.56 (4.22–4.83)	4.44 (4.22–4.83)
***P*value**	0.85	
**Age**	**< 50**	**≥ 50**
***N***	28	22
**Median score (IQR)**	4.61 (4.25–4.89)	4.56 (4.08–4.69)
***P*value**	0.29	
**Career tenure**	**< 20**	**≥ 20**
***N***	29	21
**Median score (IQR)**	4.66 (4.22–4.89)	4.44 (4.11–4.72)
***P*value**	0.35	
**Specialty**	**Generalist**	**Specialist**
***N***	9	41
**Median score (IQR)**	4.33 (4.11–4.44)	4.66 (4.22–4.89)
***P*value**	0.06	
**Type of facility**	**Hospital**	**Polyclinic**
***N***	41	9
**Median score (IQR)**	4.56 (4.22–4.83)	4.44 (4.17–4.83)
***P*value**	0.66	

### Qualitative (thematic) analysis output

The qualitative component of this study identified four themes of variables that influence the acceptability of SDM, namely the following:

1. Physician-specific (i.e., attitudes and behaviors)
2. Patient-related
3. Contextual or environmental
4. Relational or interpersonal (e.g., power or knowledge gradient)

Within the first theme, the two quotations below illustrate the perception of physicians who tend to fully engage their patients in decision-making:

P4M: “I assume that the patient wants to be fully informed and involved in the decision-making and treatment process.”

P45M: “This is my routine- no decision is made without the involvement of the patient. Clinical practice is about patient education and shared decision making.”

Also belonging to the same theme are the two examples where the physicians do not engage their patients, at all, in decision-making. They favor taking the lead, referring to manuals and other repertoire of evidences and best practices, and making the decision on behalf of their patients.

P34F: “In most cases, the decision is up to me. The patient in turn is requested to adhere to the treatment regime. I make the decision right upon undergoing the diagnosis which is based on the results. In the cases where there are multiple treatment options, I inform the patient, but I am always inclined to take the decision that I perceive as optimal for their specific cases. I do not leave it up to the patient to decide.”

P3F: “I base my decisions on evidence. I have a manual that I rely on.”

The second theme identified was “patient-related antecedents.” Under this theme, some physicians explained that their patients prefer not to be involved in decision-making related to their health. The patients’ attitude under this theme is to be blamed for lack of SDM, since patients are reluctant to take the responsibility. The quotations below highlight the findings under this theme:

P28M: “The patients either cannot take the decision or do not want to be involved. I rarely come across a patient who wants to be involved. In some cases, the patients reach me ‘spaced-out’. They tell me ‘you know better’, ‘please do not involve me’, ‘I am fed-up’, ‘make the choice that you deem fit’, and ‘take any decision’.”

P29M: “Many patients come to the clinic with the expectation of getting handled in a parental way, where the physician makes the decision on behalf of the patient and in turn bears all the responsibility. Most patients are expecting the decision to be made for them, especially patients who are from the Asian continent.”

P5F: “Patients do not want to be involved in the decision-making. They prefer that they are told what to do by the expert.”

The third theme emanating from the thematic analysis relates to environmental or contextual factors. For example, some physicians believe that there is a cultural norm, in the UAE, where taking the lead reflects that their patients trust in their credentials and credibility. In other instances, the physicians claim to minimize the patients’ involvement because there is only one (or limited) treatment option(s), and so engaging other parties would induce confusion and anxiety, complicating the dynamic as opposed to bettering it. A couple of exemplars in that regard are provided below:

P28M: “Involving patients in decision-making is not part of this culture. It does not work for this system. Sometimes we take the decision on our own; we do not even tell the patient that we need to make a decision.”

P25F: “People in this country are reluctant to learn and to bear responsibility for their decisions.”

The last theme has to do with how the physician and patient relate to one another. This theme is relational in nature where the physicians seem to be bearing the responsibility of making the decision on behalf of the patients due to the preconceived roles and responsibility of both parties. Within this realm, a group of physicians also highlight that the gradient of power and of credentials and knowledge also influence the situation. The quotations below illustrate the relational variables under this theme:

P36F: “There is a moment where the patient might not completely understand what is best for him/her. They require that additional push from our side. This push, when appropriate, gives the patient the courage that is needed to start the respective treatment.”

P47F: “There is significant knowledge asymmetry in the field of medicine. I explain a lot to my patients, not to worry them, but to increase their level of awareness. This especially applies when referring to the pros and cons.”

P23M: “When I am interacting with a lay, non-medical, and/or non-clinical patient, I prefer not to open the discussion around treatment options and personal preferences.”

## Discussion

The findings of this study revealed that physicians are supportive of engaging patients in decisions concerning their health. In fact, it is already established in the literature that physicians tend to express positive attitudes toward SDM in clinical practice [2]. However, the qualitative component of this study showed a variation of perspectives on how to position scientific evidence vis-a-vis SDM, and the margin provided to patients in partaking decisions related to their health. There are physicians who seem to favor either SDM or evidence-based medicine (EBM), and that is due to personal and professional attributes that are physician-specific which constitutes the first theme of this study’s conceptual framework. Analysis also identified three additional themes that, according to surveyed physicians, influence the degree of SDM (patient-related variables, contextual variables, and relational variables). The findings of this study concur with those published previously in regard to the unwillingness of patient to participate in decision related to their health (context/culture-specific variable) [24], their poor adherence to treatment regimens (patient-specific variable) [25], and their limited insight into condition or treatment (relational variable) [25, 26].

The group of physicians who believe that they highly engage their patients in decisions related to their health report empowering their patients and keeping them well informed throughout the treatment journey. For this group of physicians, SDM is somehow aligned with their personal value system, where for them “to do their job right” they need for the patient to play an active role throughout their treatment journey (“sharing is caring” attitude). Similar findings were reported in the primary care context [27].

Conversely, the second group of physicians mainly rely on evidence to reinforce their decisions. They believe that EBM supersedes SDM, and tend to assume that those two realms of approaching decision-making may contradict each other [24]. Those physicians rely more heavily on practice guidelines and manuals, and afford their patients narrower margins of SDM. They believe it is their own responsibility to lead the interaction and ultimately make the decisions concerning their patients’ health. This is in alignment with several studies that revealed that physicians tend to reduce their support for SDM when there is sufficient evidence in favor of one specific option [9, 28, 29].

In fact, SDM and EBM are not mutually exclusive [30, 31]. We wish to argue that the crux of patient-centered care and the optimal engagement of patients in SDM happen when the two perspectives are effectively consolidated. The essence of good clinical care stems from the use of evidence-based state-of-the-art clinical practice guidelines. However, within the boundaries of those guidelines, there are often several options that would present a window of opportunity for patients to participate in decision related to the care plan that best fits their context, preference, budget, and lifestyle. Professional learning and development programs targeting caregivers should focus on the consolidation of SDM and EBM [32, 33]. The role of professional societies includes working collaboratively with academic institutions and healthcare institutions to offer professional development programs to physicians and other healthcare providers that would strike the abovementioned balance.

Moreover, all physicians who highlighted a relatively weaker support to SDM attributed their attitudes and behaviors to variables that are outside their locus of control: patients’ stances, environment, and interpersonal dynamics. Accordingly, the practice of optimal SDM does not only require physicians to have conceptual clarity and personal conviction, it also requires the investment of resources. For example, physicians practicing SDM would need to spend more time with patients to educate them on treatment options in a language that the patients and/or their families understand [34]. Many physicians complain of heavy workloads limiting the time they could spend with patients to educate them on treatment options. Other physicians would need to be trained on communicating with patients in a language that minimizes the health literacy gap and empowers them to participate in decisions related to their health. The expansion of the care team to include health educators and experienced nurses could also decrease the time requirements on physicians while empowering other care professionals to expand their scope of practice [35, 36]. We encourage healthcare managers and leaders to “walk the talk” of patient-centered care, through the dedication of the necessary resources, making sure the institutional policies and procedures are consistent with their core values, and clearly communicating what needs to change in order to enable SDM.

In addition, improvements in the health literacy of the public through the development of educational interventions and health promotion activities would facilitate information exchange [37]. We recommend developing interventions that are designed to both improve health literacy and to educate patients on their rights (such as information leaflets and mass media campaigns). Such interventions can have a positive effect on patients’ knowledge and empowerment and in turn their health behavior and well-being. The insights that this study offers call for the development of a tool that assesses all aspects of the consultation (e.g., whether, or not, the environment is enabling, and the expectations of the patients) and not solely the attitude and behavior of the physician. It will be useful to perceive the patient-physician interaction from a linear information transmission model [38, 39] or, even better, from a constructivist communication framework perspective (i.e., models that consider how meaning is constructed by both initiators and interpreters rather than simply transmitted) [40, 41]. From that perspective, the patient will be considered as an interpreter and not solely as an information receiver, along with factoring in other aspects, for example, exploring how both parties relate to each other (e.g., language barrier or power gradient) and to the environment (e.g., physical setting and cultural norms).

This study has several shortcomings that are worth mentioning. First, it cannot be ascertained whether the physicians who disagreed to participate would have a different perspective than their participating counterparts. Second, despite assurances of the objectivity of the research team and the confidentiality of collected information, there remains a possibility of social desirability bias where physicians may be inclined to overestimate their positive views of SDM. This can explain why upon mapping the quantitative component onto the qualitative one, it turned out that a number of physicians who rated themselves highly in terms of SDM gave reasons why they do not engage their patients in decision-making. Hence, in future studies, it would be important to map the perspective of the physician with that of their patients in order to increase the validity of the generated findings. Third, while the collected information provided interesting perspectives on SDM, the overall small sample size, and limited representation across the included specialties, may have not provided the study with the necessary power to detect significant differences across various independent variables and among various specialties. It would be worthwhile for future studies to capture the perspective of a larger sample size, with statistically significant representation from all the included specialties. Finally, the external validity of the findings of this study will be specific to the care network where the investigation took place and to other similar networks. It is recommended that similar studies are carried out to investigate the degree and determinants of SDM in other contexts (e.g., public hospitals).

## Conclusion

The current study underscored a strong support among surveyed physicians for patients’ involvement in decision-making concerning their health. All physicians who seem less likely to engage in SDM attributed their attitudes and behaviors to factors that they do not have control over: patients’ stances, environment, and interpersonal dynamics. This constitutes a worthwhile opportunity to support SDM through investing in learning and development programs and ensuring the availability of the resources needed to enable SDM—be it through creating the space for the respective physician to engage their patients during the time of consultation, or empowering the patients via better educating them of their respective medical conditions. Taking such initiatives is essential to support the systems of care that aspire to become truly centered on patients.

## Data Availability

As per the IRB stipulations, the datasets used and/or analyzed during the current study are available from the lead authors on reasonable request.

## Abbreviations

EBM: Evidence-based management
SDM: Shared decision-making
SDM-Q-9: 9-item Shared Decision-Making Questionnaire
UAE: United Arab Emirates
